# Comparison of SARC-F Score among Gastrointestinal Diseases

**DOI:** 10.3390/jcm10184099

**Published:** 2021-09-10

**Authors:** Kosuke Ushiro, Hiroki Nishikawa, Masahiro Matsui, Takeshi Ogura, Toshihisa Takeuchi, Masahiro Goto, Shiro Nakamura, Kazuki Kakimoto, Takako Miyazaki, Shinya Fukunishi, Akira Asai, Hideko Ohama, Keisuke Yokohama, Hidetaka Yasuoka, Kazuhide Higuchi

**Affiliations:** 1 The Second Department of Internal Medicine, Osaka Medical and Pharmaceutical University, Takatsuki 569-8686, Japan; ushiro.1989@icloud.com (K.U.); masa1987_11_18@yahoo.co.jp (M.M.); takeshi.ogura@ompu.ac.jp (T.O.); toshihisa.takeuchi@ompu.ac.jp (T.T.); masahiro.goto@ompu.ac.jp (M.G.); saab460@gmail.com (S.N.); kazuki.kakimoto@ompu.ac.jp (K.K.); takako.miyazaki@ompu.ac.jp (T.M.); shinya.fukunishi@ompu.ac.jp (S.F.); in2108@osaka-med.ac.jp (A.A.); in2152@osaka-med.ac.jp (H.O.); hammer_0906@yahoo.co.jp (K.Y.); yh0403.4351@gmail.com (H.Y.); kazuhide.higuchi@ompu.ac.jp (K.H.); 2 The Premier Departmental Research of Medicine, Osaka Medical and Pharmaceutical University, Takatsuki 569-8686, Japan

**Keywords:** SARC-F, gastrointestinal disease, sarcopenia, predictor

## Abstract

SARC-F is a screening tool for sarcopenia. We sought to compare the SARC-F scores of patients with different gastrointestinal diseases (*n* = 1282 (762 males): upper gastrointestinal disease (UGD, *n* = 326), lower gastrointestinal disease (LGD, *n* = 357), biliary and pancreatic disease (BPD, *n* = 416), and liver disease (LD, *n* = 183)). Factors associated with SARC-F ≥4 points (highly suspicious of sarcopenia) were also examined. The median age was 71 years. Patients with SARC-F ≥4 points were found in 197 (15.4%). Advanced cancer was found in 339 patients (26.4%). The proportion of SARC-F ≥4 points in groups of UGD, LGD, BPD, and LD were 17.5% (57/326) in UGD, 12.0% (43/357) in LGD, 17.3% (72/416) in BPD, and 13.7% (25/183) in LD, respectively (overall *p* = 0.1235). In patients with and without advanced cancer, similar tendencies were observed. In the multivariate analysis, age (*p* < 0.0001), gender (*p* = 0.0011), serum albumin (*p* < 0.0001), lymphocyte count (*p* = 0.0019), C reactive protein (*p* = 0.0197), and the presence of advanced cancer (*p* = 0.0424) were significant factors linked to SARC-F ≥4 points. In patients with advanced cancer, SARC-F scores correlated well with their Glasgow prognostic scores. In conclusion, sarcopenia in gastrointestinal diseases may be affected not by disease type (i.e., the primary origin of the disease) but by aging, nutritional condition, inflammatory condition, and cancer burden.

## 1. Introduction

Sarcopenia is a clinical symptom characterized by progressive and generalized loss of muscle mass and muscle functional weakness that results in frailty, cachexia, severe infection, osteoporosis, and thereby all-cause mortality [1,2]. Inactivity in daily life, bedridden status, malnutrition, advanced cancer-bearing conditions, and chronic inflammatory conditions frequently seen in patients with gastrointestinal diseases are typical clinical features leading to sarcopenia [1,2,3,4,5,6,7,8,9]. Alterations in nutritional metabolism, nutritional requirements, and reduced dietary intakes are also often encountered in patients with gastrointestinal diseases [8], and sarcopenia in patients with gastrointestinal diseases can be associated with worse patient QOL, poorer prognosis, and more expensive health care costs [8,10]. Gastrointestinal diseases are representative of secondary sarcopenia caused by the disease itself [10]. Thus, sarcopenia in gastrointestinal diseases is an increasing problem in recent years and can be one of the major concerns in clinical settings.

SARC-F is a screening tool for sarcopenia and is a questionnaire consisting of five questions [11,12]. Patients are asked to rate their Strength (S; weakness), Assistance walking (A; with or without walking aid), Rising from a chair (R; rising from a chair), Climbing stairs (C; climbing stairs), and Falls (F; falling) on a scale of 0 to 2 from “not difficult at all” to “very difficult,” and the total score (out of 10) is calculated. The recommended cut-off value of the SARC-F score is 4 points [11]. The SARC-F score is closely associated with grip strength, physical function, and patient QOL [13,14]. Patients with SARC-F score ≥4 points are determined to be highly suspicious of sarcopenia [11]. In a recent report, SARC-F has been found to be useful for detecting frailty as well as sarcopenia in elderly people [15]. Current Asian Working Group for Sarcopenia (AWGS) guidelines and European Working Group for Sarcopenia in Older People (EWGSOP) guidelines recommend the use of SARC-F as a first screening tool for sarcopenia [16,17], although its low to moderate sensitivity for sarcopenia may be a concern [18,19,20]. A previous meta-analysis reported that the pooled results of sensitivity and specificity for sarcopenia were 0.21 and 0.90 [12]. One way to compensate for the low sensitivity of SARC-F is to add the calf circumference [16].

To the best of our knowledge, however, there have been no reports comparing SARC-F scores by different gastrointestinal diseases (upper gastrointestinal disease (UGD), lower gastrointestinal disease (LGD), biliary and pancreatic disease (BPD), and liver disease (LD)). This clinical research question may be important in daily clinical practice. Therefore, in the current study, we aimed to elucidate these issues.

## 2. Patients and Methods

### 2.1. Patients

Our hospital includes one of the leading high-volume centers for gastrointestinal diseases in Japan. Gastrointestinal diseases considered in this study are UGD, LGD, BPD, and LD. UGD included patients with early or advanced esophageal cancer, early or advanced gastric cancer, upper gastrointestinal bleeding, and others. LGD patients included those with early or advanced colorectal cancer, colon polyp, inflammatory bowel diseases, lower gastrointestinal bleeding, and others. Patients with BPD included those with early or advanced hepatobiliary cancer, early or advanced pancreatic cancer, common bile duct stone, acute or chronic pancreatitis, acute cholecystitis, pancreatic cystic diseases, and others. LD included patients with early or advanced hepatic cancer, acute hepatitis, acute liver injury, refractory ascites due to liver diseases, and others. The data for each patient have been consecutively recorded in our database. In accordance with principle, all hospitalized patients were asked to fill out the SARC-F questionnaire at the time of hospitalization, except for those who were unable to fill out the questionnaire due to dementia or other reasons. Between May 2020 and May 2021, there was a total of 1282 Japanese gastrointestinal disease patients with data for SARC-F scores in our database. All patients were admitted for the treatment or diagnosis of gastrointestinal diseases. Baseline variables included were the following: age (years), gender, body mass index (BMI, kg/m^2^), serum albumin level (g/dL), total lymphocyte count (/μL), estimated glomerular filtration rate (eGFR, mL/min/1.73 m^2^), and C reactive protein (CRP, mg/dL). All personal information was carefully protected.

### 2.2. SARC-F Score and Our Analysis

As mentioned earlier, the SARC-F for each patient was calculated. First, SARC-F scores and the proportion of patients with SARC-F ≥4 points were compared according to patients’ diseases (UGD, LGD, BPD, and LD). Next, factors associated with SARC-F ≥4 points (highly suspicious of sarcopenia) were examined using univariate and multivariate analyses. Advanced cancer was determined based on NCCN Clinical Practice Guidelines in Oncology using radiological findings (TNM stage classification, stage III or VI).

We received ethical approval from the ethics committee of the Osaka Medical and Pharmaceutical University Hospital (approval number 2021-027). The protocol in this study strictly followed all regulations of the 1975 Declaration of Helsinki.

### 2.3. Statistical Considerations

In terms of continuous parameters, the Student’s *t*-test or the Mann–Whitney U test was applied to perform two-group comparisons as appropriate after the confirmation of normality, and the ANOVA or the Kruskal–Wallis test was applied to perform multiple-group comparisons as appropriate after the confirmation of normality. Data for continuous parameters were presented using their median value (interquartile range, IQR). In terms of categorical parameters, the Pearson χ^2^ test was applied to estimate the between-group differences. Multivariate logistic regression analysis linked to SARC-F ≥4 points was also performed to identify independent factors. In the multiple comparison, the cutoff point for statistical significance was set at *p* = 0.05 using JMP ver. 15 (SAS Institute Inc., Cary, NC, USA). For the comparisons between each group of two, the Bonferroni correction was used as a method to adjust for type I error. A total of 6 tests were performed for the comparisons between each of two groups in the four-group comparison, and thus the significance level was set at *p* < 0.05 divided by 6. In the comparisons between each group in the three-group comparison, *p* < 0.05 divided by 3 was set as the significance level because the test was conducted 3 times.

## 3. Results

### 3.1. Patient Baseline Characteristics

**Figure 1:**
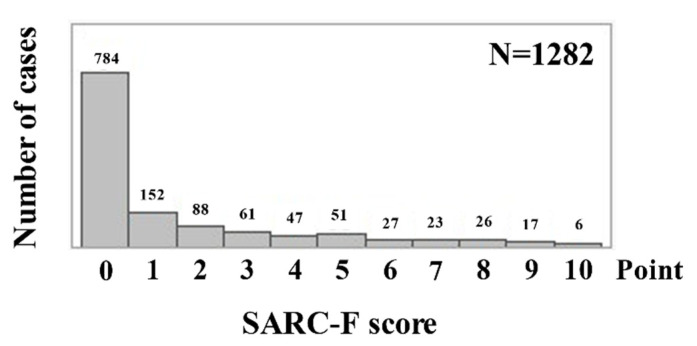
Number of patients according to SARC-F score.

Baseline characteristics for all cases (*n =* 1282, 762 males and 520 females, median (IQR) age = 71 (61–78) years) are presented in Table 1. The median (IQR) BMI was 22.1 (19.6–24.3) kg/m^2^. The median (IQR) serum albumin level was 3.8 (3.3–4.1) g/dL. UGD was found in 326 patients (advanced cancer cases of UGD: 108 (33.1%)), LGD in 357 (advanced cancer cases of LGD: 82 (23.0%)), BPD in 416 (advanced cancer cases of BPD: 93 (22.3%)), and LD in 183 (advanced cancer cases of LD: 56 (30.6%)). Overall, advanced cancer cases were noted in 339 patients (26.4%). The number of cases according to the SARC-F scores is shown in Figure 1. Patients’ SARC-F of 0 points were observed in 784 cases (61.2%), SARC-F of 1 point in 152 (11.9%), SARC-F of 2 points in 88 (6.9%), SARC-F of 3 points in 61 (4.8%), and SARC-F of ≥4 points in 197 (15.4%).

### 3.2. Comparison of Baseline Characteristics among 4 Groups of UGD, LGD, BPD, and LD

The comparisons of baseline characteristics among four groups of UGD, LGD, BPD, and LD are shown in Table 2. In terms of age (*p* < 0.0001), gender (*p* = 0.0004), BMI (*p* = 0.0046), and CRP (*p* = 0.0019), overall differences among the four groups were identified with significance. Notably, the proportion of males in the UGD group was the highest among the four groups (68.7%, 224/326), and the median age in the LGD group was the lowest among the four groups (68 years).

### 3.3. SARC-F Score According to the Diseases (UGD, LGD, BPD, and LD)

**Figure 2:**
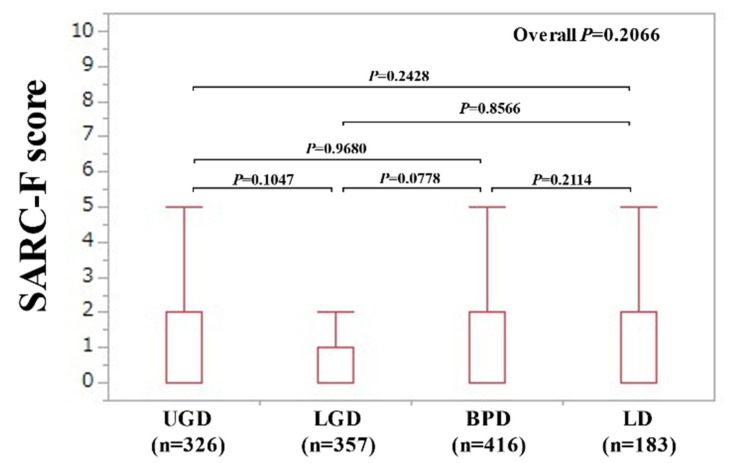
SARC-F score according to the diseases (upper gastrointestinal disease (UGD), lower gastrointestinal disease (LGD), biliary and pancreatic disease (BPD), and liver disease (LD)).

The median (IQR) SARC-F scores in the disease groups were the following: 0 (0–2) in UGD, 0 (0–1) in LGD, 0 (0–2) in BPD, and 0 (0–2) in LD (*p* values: UGD vs. LGD, *p* = 0.1047; UGD vs. BPD, *p* = 0.9680; UGD vs. LD, *p* = 0.2428; LGD vs. BPD, *p* = 0.0778; LGD vs. LD, *p* = 0.8566; BPD vs. LD, *p* = 0.2114; overall *p* = 0.2066) (Figure 2).

### 3.4. Proportion of Patients with SARC-F ≥4 Points According to Disease (UGD, LGD, BPD, and LD)

**Figure 3:**
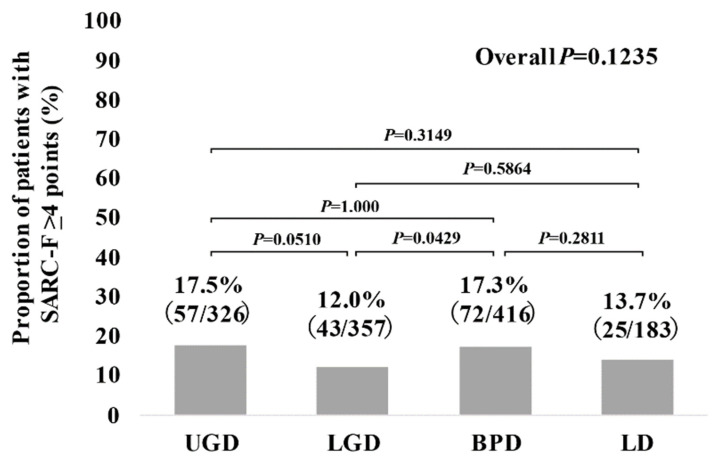
The proportion of patients with SARC-F ≥4 points according to the diseases (UGD, LGD, BPD, and LD).

The proportions of SARC-F ≥4 points in the disease groups were 17.5% (57/326) in UGD, 12.0% (43/357) in LGD, 17.3% (72/416) in BPD, and 13.7% (25/183) in LD (*p* values: UGD vs. LGD, *p* = 0.0510; UGD vs. BPD, *p* = 1.000; UGD vs. LD, *p* = 0.3149; LGD vs. BPD, *p* = 0.0429; LGD vs. LD, *p* = 0.5864; BPD vs. LD, *p* = 0.2811; overall *p* = 0.1235) (Figure 3).

### 3.5. SARC-F Score According to Disease (UGD, LGD, BPD, and LD) in Patients with and without Advanced Cancer Cases

**Figure 4:**
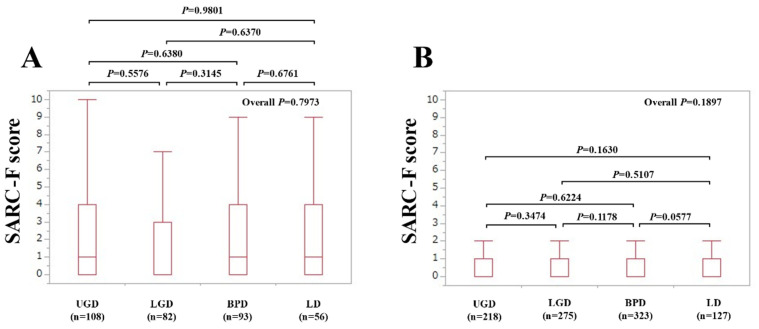
(**A**) SARC-F scores according to the diseases (UGD, LGD, BPD, and LD) in patients with advanced cancer (*n =* 339). (**B**) SARC-F scores according to the diseases (UGD, LGD, BPD, and LD) in patients without advanced cancer (*n =* 943).

The median (IQR) SARC-F scores in the groups of UGD, LGD, BPD, and LD patients with advanced cancer (*n =* 339) were the following: 1 (0–4) in UGD (*n =* 108), 0 (0–3) in LGD (*n =* 82), 1 (0–4) in BPD (*n =* 93), and 1 (0–4) in LD (*n =* 56) (*p* values: UGD vs. LGD, *p* = 0.5576; UGD vs. BPD, *p* = 0.6380; UGD vs. LD, *p* = 0.9801; LGD vs. BPD, *p* = 0.3145; LGD vs. LD, *p* = 0.6370; BPD vs. LD, *p* = 0.6761; overall *p* = 0.7973) (Figure 4A).

The median (IQR) SARC-F scores in the groups of UGD, LGD, BPD, and LD patients without advanced cancer (*n =* 943) were the following: 0 (0–1) in UGD (*n =* 218), 0 (0–1) in LGD (*n =* 275), 0 (0–1) in BPD (*n =* 323), and 0 (0–1) in LD (*n =* 127) (*p* values: UGD vs. LGD, *p* = 0.3474; UGD vs. BPD, *p* = 0.6224; UGD vs. LD, *p* = 0.1630; LGD vs. BPD, *p* = 0.1178; LGD vs. LD, *p* = 0.5107; BPD vs. LD, *p* = 0.0577; overall *p* = 0.1897) (Figure 4B).

### 3.6. Proportion of Patients with SARC-F ≥4 Points According to Disease (UGD, LGD, BPD, and LD) in Patients with and without Advanced Cancer Cases

**Figure 5:**
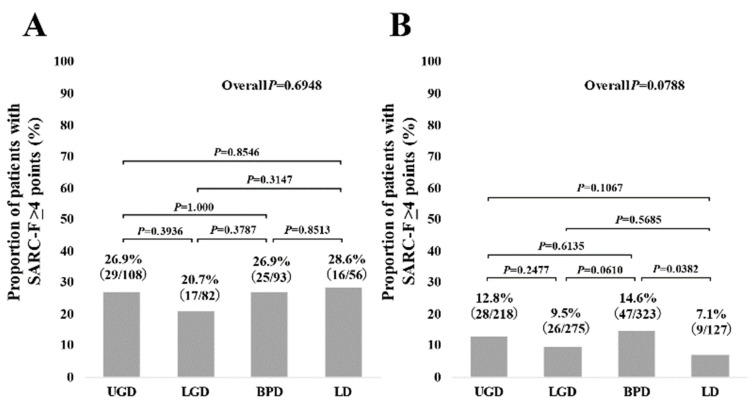
(**A**) The proportions of patients with SARC-F ≥4 points according to the diseases (UGD, LGD, BPD, and LD) in patients with advanced cancer (*n =* 339). (**B**) The proportion of patients with SARC-F ≥4 points according to the diseases (UGD, LGD, BPD, and LD) in patients without advanced cancer (*n =* 943).

The proportions of SARC-F ≥4 points in the groups of UGD, LGD, BPD, and LD in patients with advanced cancer were 26.9% (29/108) in UGD, 20.7% (17/82) in LGD, 26.9% (25/93) in BPD, and 28.6% (16/56) in LD (*p* values: UGD vs. LGD, *p* = 0.3936; UGD vs. BPD, *p* = 1.000; UGD vs. LD, *p* = 0.8546; LGD vs. BPD, *p* = 0.3787; LGD vs. LD, *p* = 0.3147; BPD vs. LD, *p* = 0.8513; overall *p* = 0.6948) (Figure 5A).

The proportions of SARC-F ≥4 points in groups of UGD, LGD, BPD, and LD in patients without advanced cancer were 12.8% (28/218) in UGD, 9.5% (26/275) in LGD, 14.6% (47/323) in BPD, and 7.1% (9/127) in LD (*p* values: UGD vs. LGD, *p* = 0.2477; UGD vs. BPD, *p* = 0.6135; UGD vs. LD, *p* = 0.1067; LGD vs. BPD, *p* = 0.0610; LGD vs. LD, *p* = 0.5685; BPD vs. LD, *p* = 0.0382; overall *p* = 0.0788) (Figure 5B).

### 3.7. Univariate and Multivariate Analyses of Factors Associated with SARC-F ≥4 Points

In the univariate analysis, age (*p* < 0.0001), gender (*p* = 0.0074), BMI (*p* = 0.0103), serum albumin (*p* < 0.0001), total lymphocyte count (*p* < 0.0001), eGFR (*p* = 0.0026), CRP (*p* < 0.0001), and the presence of advanced cancer (*p* < 0.0001) were significant factors linked to SARC-F ≥4 points (Table 3). Age (*p* < 0.0001), gender (*p* = 0.0011), serum albumin (*p* < 0.0001), total lymphocyte count (*p* = 0.0019), CRP (*p* = 0.0197), and the presence of advanced cancer (*p* = 0.0424) were independent factors linked to patients having SARC-F ≥4 points in the multivariate logistic regression analysis (Table 4). Hazard ratios and 95% confidence intervals in each factor are shown in Table 4.

### 3.8. Proportion of SARC-F ≥4 Points and SARC-F Score According to Glasgow Prognostic Score (GPS) in Patients with Advanced Cancer

**Figure 6:**
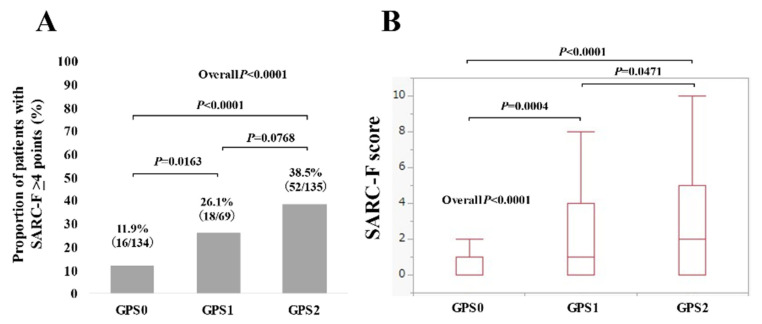
(**A**) The proportion of patients with SARC-F ≥4 points according to Glasgow prognostic scores (GPSs). (**B**) SARC-F scores according to GPSs.

Serum albumin level and CRP were independent predictors linked to SARC-F ≥4 points. Thus, we compared the proportion of patients with SARC-F ≥4 points and SARC-F scores according to GPSs in patients with advanced cancer (*n* = 338, missing data (*n* = 1)) [21]. GPS is determined by serum albumin level (cutoff value = 3.5 g/dL) and CRP level (1.0 mg/dL) and is a well-validated prognostic system in patients with advanced cancer [22,23]. There were 134 patients with GPS 0 (serum albumin ≥ 3.5 g/dL and CRP ≤ 1.0 mg/dL), 69 with GPS 1 (serum albumin ≥ 3.5 g/dL and CRP > 1.0 mg/dL, or serum albumin < 3.5 g/dL and CRP ≤ 1.0 mg/dL), and 135 with GPS 2 (serum albumin < 3.5 g/dL and CRP >1.0 mg/dL). The proportion of patients with SARC-F ≥4 points (11.9% (16/134) in GPS 0, 26.1% (18/69) in GPS 1, and 38.5% (52/135) in GPS 2) was stratified well by GPSs (*p* values: GPS 0 vs. 1, *p* = 0.0163; GPS 1 vs. 2, *p* = 0.0768; GPS 0 vs. 2, *p* < 0.0001; overall *p* < 0.0001) (Figure 6A). The median (IQR) SARC-F scores in patients with GPS 0, 1 and 2 were 0 (0–1) in GPS 0, 1 (0–4) in GPS 1, and 2 (0–5) in GPS 2 (*p* values: GPS 0 vs. 1, *p* = 0.0004; GPS 1 vs. 2, *p* = 0.0471; GPS 0 vs. 2, *p* < 0.0001; overall *p* < 0.0001) (Figure 6B).

## 4. Discussion

More than three decades have passed since Rosenberg first proposed the concept of sarcopenia in 1989 [24]. In the field of public health, sarcopenia has been attracting much caution these days due to its close association with prognosis (e.g., falls, fracture, infection, frailty and survival) [1,2,3,4,5,6,7,10]. To diagnose sarcopenia, it is necessary to assess muscle mass, but computed tomography and bioelectrical impedance analysis for muscle mass assessment are often not available in small clinics [16]. Yang et al. reported a very good area under the receiver-operating characteristic curve of 0.86 and 0.90 for males and females, respectively, using the recommended cutoff value of SARC-F (i.e., 4 points) for the diagnosis of sarcopenia [25]. Thus, SARC-F seems to be very useful for the assessment of sarcopenia, although the sensitivity of SARC-F for sarcopenia is low, and, as described above, the international guidelines (AWGS guidelines and EWGSOP guidelines) also recommend the use of SARC-F as the first step of sarcopenia screening [12,16,17,25]. However, as far as we are aware, no research can be found comparing SARC-F scores grouped by gastrointestinal diseases. Our study appears to be the first report to elucidate these issues. A large number of cohorts (*n =* 1282) is one of the major strengths of this study.

Overall, SARC-F scores and the frequency of patients with SARC-F ≥4 points were almost similar across the gastrointestinal diseases (UGD, LGD, BPD, and LD), although the baseline characteristics among the four groups were different in our results. In all the two-group comparisons (Figure 2, Figure 3, Figure 4 and Figure 5), no significant differences were found after the Bonferroni correction. In addition, gastrointestinal disease type was not linked to SARC-F ≥4 points as a significant factor, while age, gender, serum albumin, lymphocyte count, CRP, and the presence of advanced cancer were significant factors linked to SARC-F ≥4 points in the multivariate analysis. Again, disease type was not significant either in the univariate or multivariate analyses. We could not find sufficient evidence to support the association between sarcopenia and the anatomical categories of gastrointestinal diseases. Therefore, while sarcopenia in gastrointestinal diseases may be unaffected by gastrointestinal disease type (i.e., the primary origin of the disease), it is affected by aging, nutritional condition, inflammatory condition, and cancer burden. On the other hand, in our study, there were 197 patients (15.4%) with SARC-F ≥4 points. Patients with SARC-F ≥4 points are very likely to be diagnosed with sarcopenia [12,19]. In a large survey (*n =* 4811) of elderly Japanese subjects, the prevalence of sarcopenia was shown to be 7.5% [26]. The prevalence of sarcopenia in large studies with more than 1000 subjects is 6–12% [26,27,28]. The disease burden itself causing secondary sarcopenia may account for the difference between studies [7].

The proportion of patients with SARC-F ≥4 points in the LGD group was the lowest (12.0%) among the four groups. This is probably due to the LGD patients having the youngest ages (median age = 68 years). The LGD group included 66 patients with inflammatory bowel diseases (18.5%), such as ulcerative colitis and Crohn’s disease, the presence of which corresponded with relatively smaller patient ages. The median (IQR) SARC-F score in these 66 patients was 0 (0–0), and the proportion of patients with SARC-F ≥4 points was 6.1% (4/66). Sarcopenia contemporaneous with gastrointestinal diseases can be complicated by primary factors due to aging and secondary factors due to the disease burden itself [2,7,10]. Nutritional aspects and inflammatory aspects are both relevant for the incidence of sarcopenia [29]. Systemic inflammation, as reflected by CRP level, can activate ubiquitin-proteasome pathways, leading to muscle proteolysis [30]. Inflammatory cytokines such as IL-6 and TNFα can also cause insulin resistance, apoptosis, and dysfunction of mitochondria, which are involved in energy production, excessive production of reactive oxygen species, and the acceleration of muscle protein breakdown to eventually result in sarcopenia [31,32,33,34,35,36,37]. Myokines, such as IGF-1, with the role of promoting muscle protein synthesis, and myostatin, with the role of suppressing muscle protein synthesis, are also important for muscle protein homeostasis [38,39,40].

GPS is a widely accepted prognostic system in patients with advanced cancer [22,23]. In our data, GPSs correlated well with SARC-F scores and the proportion of patients with SARC-F ≥4 points, which indicates the usefulness of reviewing SARC-F scores in patients with advanced cancer. Cachexia staging scores in patients with advanced cancer reported by Zhou et al. included SARC-F scores [41]. On the other hand, gender was an independent factor linked to SARC-F ≥4 points in the multivariate analysis. The median (IQR) SARC-F scores in males and females were 0 (0–1) and 0 (0–2) (*p* < 0.0001), respectively. The reason for these, however, is unclear, and further examinations regarding the impact of gender difference on SARC-F scores will be required.

Several limitations must be mentioned in the present study. Firstly, our study was a cross-sectional study at a single institution and was retrospective in nature. Secondly, baseline characteristics among the four groups were slightly different, potentially creating bias. Thirdly, our patient population, manifesting malignancies, benign tumors, inflammatory diseases, and infections, was highly heterogeneous and thus also leads to bias. Fourthly, SARC-F is a self-reported questionnaire for possible sarcopenia, and data for the number of patients with definite sarcopenia were not included in our current analysis. Caution should therefore be paid for the interpretation of our data. Despite the limitations, our study results demonstrated that not the gastrointestinal disease type, but rather aging, poorer nutritional condition, a severer inflammatory condition, and cancer status may be associated with higher SARC-F scores.

In conclusion, there was not sufficient evidence to support the association between sarcopenia and separate anatomical categories of gastrointestinal disease. Sarcopenia concurrent with gastrointestinal diseases may be affected not by disease type (i.e., the primary origin of the disease) but rather aging, nutritional condition, inflammatory condition, and cancer burden.

## Figures and Tables

**Table 1:** Baseline characteristics (*n =* 1282).

	Number or Median (IQR)
Age (years)	71 (61–78)
Gender, male/female	762/520
Body mass index (kg/m^2^)	22.1 (19.6–24.3)
Serum albumin (g/dL)	3.8 (3.3–4.1)
Total lymphocyte count (/μL)	1270 (897–1714)
eGFR (mL/min/1.73 m^2^)	67 (54–81)
C reactive protein (mg/dL)	0.22 (0.06–1.63)
Breakdown of diseases (number and % of advanced cancer)	
Upper gastrointestinal disease (UGD)	326 (108, 33.1%)
Lower gastrointestinal disease (LGD)	357 (82, 23.0%)
Biliary and pancreatic disease (BPD)	416 (93, 22.3%)
Liver disease (LD)	183 (56, 30.6%)

eGFR, estimated glomerular filtration rate; IQR, interquartile range.

**Table 2:** A comparison of baseline characteristics among 4 groups of UGD, LGD, BPD, and LD.

	UGD (*n =* 326)	LGD (*n =* 357)	BPD (*n =* 416)	LD (*n =* 183)	*p* Value
Age	72 (64–78)	68 (52–76)	73 (66–80)	72 (65–78)	<0.0001
Gender, male (%)	224 (68.7)	189 (52.9)	240 (57.7)	109 (59.6)	0.0004
BMI (kg/m^2^)	22 (19.5–24.1)	22.1 (19.2–24.5)	21.8 (19.5–24.2)	23.1 (20.3–25.5)	0.0046
Serum albumin (g/dL)	3.8 (3.3–4.1)	3.8 (3.2–4.1)	3.9 (3.4–4.2)	3.7 (3.1–4.1)	0.0662
Total lymphocyte count (/μL)	1269 (932–1752)	1266 (911–1741)	1293 (870–1702)	1224 (787–1576)	0.5622
eGFR (mL/min/1.73 m^2^)	66 (53–79.3)	69 (54–83.3)	67.5 (55.8–81.3)	67 (54–80)	0.0843
CRP (mg/dL)	0.14 (0.05–0.71)	0.25 (0.06–1.76)	0.27 (0.07–2.4)	0.22 (0.08–1.53)	0.0019

Data are presented as a number or the median value (interquartile range). UGD, upper gastrointestinal disease; LGD, lower gastrointestinal disease; BPD, biliary and pancreatic disease; LD, liver disease; BMI, body mass index; eGFR, estimated glomerular filtration rate; CRP, C reactive protein.

**Table 3:** Univariate analysis of factors linked to SARC-F ≥4 points.

	SARC-F ≥ 4 Points (*n =* 197)	SARC-F < 4 Points (*n =* 1085)	*p* Value
Age (years)	78 (72–84)	70 (59–77)	<0.0001
Gender, male/female	100/97	662/423	0.0074
BMI (kg/m^2^)	21.4 (19–24.1)	22.1 (19.8–24.5)	0.0103
Serum albumin (g/dL)	3.3 (2.8–3.7)	3.9 (3.4–4.2)	<0.0001
Lymphocyte count	975 (639–1341)	1329 (944–1778)	<0.0001
eGFR (mL/min/1.73 m^2^)	59.5 (41–78)	68 (55–81)	<0.0001
CRP (mg/dL)	0.79 (0.17–5.78)	0.17 (0.06–1.15)	<0.0001
Type of disease, UGD/LGD/BPD/LD	57/43/72/25	269/314/344/158	0.1235
Advanced cancer, yes/no	87/110	252/833	<0.0001

Data are presented as a number or median the value (IQR). BMI, body mass index; eGFR, estimated glomerular filtration rate; CRP, C reactive protein; UGD, upper gastrointestinal disease; LGD, lower gastrointestinal disease; BPD, biliary and pancreatic disease; LD, liver disease.

**Table 4:** Multivariate analysis of factors linked to SARC-F ≥4 points.

	Multivariate Analysis		
	Hazard Ratio	95% CI	*p* Value
Age (per one year)	1.067	1.047–1.088	<0.0001
Gender (female)	1.796	1.263–2.553	0.0011
BMI (per one kg/m^2^)	0.980	0.933–1.028	0.4006
Serum albumin (per one g/dL)	0.416	0.305–0.567	<0.0001
Lymphocyte count (per one /μL)	0.9995	0.9992–0.9998	0.0019
eGFR (per one mL/min/1.73 m^2^)	0.999	0.992–1.007	0.8807
CRP (per one mg/dL)	1.041	1.007–1.077	0.0197
Advanced cancer	1.481	1.014–2.163	0.0424
Type of disease			
UGD	1.400	0.861–2.276	0.1750
LGD	1.000	Reference	
BPD	1.082	0.674–1.737	0.7450
LD	1.324	0.724–2.422	0.3626

BMI, body mass index; eGFR, estimated glomerular filtration rate; CRP, C reactive protein; UGD, upper gastrointestinal disease; LGD, lower gastrointestinal disease; BPD, biliary and pancreatic disease; LD, liver disease; CI, confidence interval.

## Data Availability

Data available on request due to restrictions eg privacy or ethical.

## Abbreviations

UGD, upper gastrointestinal disease; LGD, lower gastrointestinal disease; BPD, biliary and pancreatic disease; LD, liver disease; BMI, body mass index; CRP, C reactive protein; IQR, interquartile range; eGFR, estimated glomerular filtration rate; AWGS, Asian Working Group for Sarcopenia; EWGSOP, European Working Group for Sarcopenia in Older People; GPS, Glasgow prognostic score.
